# Making co-creation operational: A RECONECT seven-steps-pathway and practical guide for co-creating nature-based solutions

**DOI:** 10.1016/j.mex.2023.102495

**Published:** 2023-12-09

**Authors:** Dushkova Diana, Kuhlicke Christian

**Affiliations:** a Department Biodiversity Conservation and Socio-Ecological Systems, Helmholtz Centre for Environmental Research – UFZ, Germany; b Department Urban and Environmental Sociology, Helmholtz Centre for Environmental Research – UFZ, Permoserstr. 15, Leipzig 04318, Germany

**Keywords:** Co-creation, Nature-based solutions, Participatory approach, Stakeholder's engagement, Hydro-meteorological risk reduction, This is a practical guidance on how to establish and operationalize co-creation process of nature-based solutions (NBS) according to the RECONECT project method / strategy

## Abstract

Co-creation as a practice of collaborative product or service development is not a novel concept, however its application in the field of nature-based solutions (NBS) requires a certain level of knowledge, expertise and capacity building to ensure a shared understanding and collaborative dialogue. NBS are defined as cost-effective and ecosystem-based solutions to solving sustainability challenges and climate-change pressures through embedding a more citizen-oriented engagement within its implementation. Although some co-creation principles and guidelines are scientifically well elaborated, only a few of them was put into practice. Thus there is still a need for making them clearer and more feasible to a broad range of stakeholders from the non-scientific community. This problem is mostly caused by the lack of easy-to use framework / strategy for organizing a co-creation and selecting the appropriate co-creation activity for the certain purpose of the NBS realization process. It includes making a right decision on what particular tools, how and with what groups of stakeholders can be applied in a certain case and by the availability of particular resources. For this purpose, a stepwise pathway/guide on how participatory approach can be incorporated into the whole process of NBS co-creation was developed within the RECONECT project. The main innovative contribution of this work is to propose the following ready-to-use solutions:

• detailed seven-steps-co-creation pathway and practical recommendations for NBS design and implementation;
• multidimensional and comprehensive decision-making matrix consisting of 88 tools for selecting the most suitable solution (tool) according to the particular co-creation goal, available resources and capacities;
• additional resources to facilitate and operationalise the co-creation process at each stage of NBS development, offered in the form of a Toolbox.

## Graphical abstract

**Figure:**
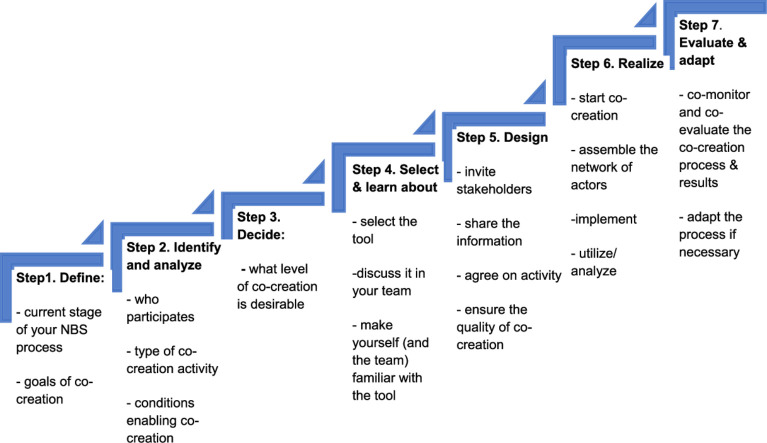

Specifications table

**Table:** 

Subject Area:	Environmental Science
More specific subject area:	*Environmental Sociology*
Method name:	This is a practical guidance on how to establish and operationalize co-creation process of nature-based solutions (NBS) according to the RECONECT project method / strategy
Name and reference of original method:	Inspiration from theory on co-creation and review of existing guidance on stakeholder mapping and engagement developed by: • Dam and Siagn [1] Design Thinking: Getting Started with Empathy. Interaction Design Foundation. Available online: https://www.interaction-design.org/literature/article/design-thinking-getting-started-with-empathy (accessed on 6 May 2022). • Drajic et al. [2] User Engagement for Large Scale Pilots in the Internet of Things. 14th International Conference on Advanced Technologies, Systems and Services in Telecommunications (TELSIKS), 2019, 46–53, doi: 10.1109/TELSIKS46999.2019.9002017. • Morello et al. [3] Guidance on co-creating nature-based solutions PART II—running CLEVER Action Labs in 16 steps. Deliverable 1(1):6. Available online: https://clevercities.eu/fileadmin/user_upload/Resources/D1.1_Theme_5_Co-creation_framework_FPM_12.2018.pdf (accessed on 21 May 2022). • Service Design Tools [4]. The open collection of tools and tutorials that helps dealing with complex design challenges. Available online: http://www.servicedesigntools.org/ (accessed on 7 February 2022). • UNaLab. UNaLab Co-Creation Toolkit [5] Available online: https://unalab.enoll.org/ (accessed on 16 May 2022). • van der Have et al. [6] A practical guide to using co-production for nature-based solutions. Connecting Nature project. Available online: https://connectingnature.eu/sites/default/files/downloads/CN-Co-production_for_NBS-Guidebook-MidRes.pdf (accessed on 06 May 2022) • Van der Pijl P. et al. Design a better business: New tools, skills and mindsets [7] Available online: https://designabetterbusiness.com/ (accessed on 12 February 2022) • Wippoo, M.; van Dijk, D. BigPicnic D5.1: Toolkit on Co-Creation Process; [8]; Available online: https://ec.europa.eu/research/participants/documents/downloadPublic?documentIds=080166e5c390a64e&appId=PPGMS (accessed on 10 July 2022).
Resource availability:	*RECONECT co-creation strategy (incl. detailed co-creation pathway to NBS and Toolboxes) in form of RECONECT Manual for practitioners*[9]*is currently in the process of being made publicly available and accessible online for a broad range of stakeholders, actors and researchers interested in the process of co-creating NBS. It will be freely accessible at the project website:*www.reconect.eu*and website of UFZ:*www.ufz.de

## Background

Co-creation is understood as a collaborative approach which allows stakeholders to be involved in designing and building more inclusive and sustainable mechanisms for change. This approach is used in a wide range of fields related to nature-based solutions (NBS) [[3], [10], [11]]. Defined by the European Commission [12] as cost-effective solutions that are inspired and supported by nature, NBS help in building resilience by providing benefits across economic, environmental, and social pillars. NBS are considered to be a new design strategy and planning tool for the building of resilient, sustainable, livable and healthy cities and communities [13]. The main purpose of co-creation here is to enhance the awareness and knowledge of society and stakeholders regarding NBS, to balance interests, benefits and responsibilities between relevant stakeholders, to focus attention on user needs. Co-creation also allows to make the whole process – from planning to implementation of NBS and evaluation of its impact – transparent and inclusive, accelerating the need for capacity building in public administration towards an effective shared governance [[6], [14],^15^]. Although specific methodologies vary, co-creation and participatory approaches involve a diverse group of stakeholders to solve tasks such as setting research objectives, gathering and processing data, interpreting results, implementing solutions as well as monitoring and evaluating the impact and benefits provided by NBS [[3], [16], [17], [18], [19]].

Several EC projects dealing with NBS have already used the co-creation approach and developed their own strategy / toolkits for applying it in urban context (e.g. CONNECTING Nature, PHUSICOS, OPERANDUM, CLEVER Cities, Nature4Cities, NATURVATION, UNaLab, etc.). Although they suggested a variety of methods, principles and tools for co-creation as well as strategies for stakeholders’ engagement in various stages of the NBS process [[3], [5], [6], [14], [15], [18], [20], [21]], we argue that a specific algorithm and step-by-step guide/pathway are needed for a practical application not only in urban areas, but also with regard to operationalizing the existing knowledge on participatory approach to NBS at other scales. Considering the above-mentioned, one of the ambitions of the RECONECT project is to go beyond previous studies by providing an easy-to-use strategy/pathway for the co-creation process in order to stimulate a new culture of co-creation, expanding the scale from urban to ‘land use planning’ that links the reduction of hydro-meteorological risk with local and regional development objectives in a sustainable and financially viable way. During our work on the project, we often received requests from the RECONECT partners for practical guidelines on how to design and implement participatory processes for co-creating NBS. Usually, it is difficult to find practical information for participatory methods, and although for some methods or tools there is a considerable documentation available, it is often rather academic. Also, from our academic perspective and from our own experience of working in different NBS related projects we can state: a lot of practical knowledge is being developed but little is put on paper, or if published, it is mostly available only in the academic world. Thus, the main purpose of this paper is to provide an easy-to-use methodical approach which can explain to the NBS practitioners how to make co-creation operational using the RECONECT seven-steps-pathway and practical guide, including a matrix of tools for co-creating NSB. By providing practical guidance on how to design, implement and facilitate the co-creation of NBS, the paper:

(a) sets out particular activities (steps) for co-creating NBS using a participatory approach which involves a diversity of stakeholders;
(b) offers practical recommendations for its design and implementation;
(c) presents a decision-making matrix of tools which helps in finding appropriate co-creation tools according to the specific co-creation goal, resources and capacities;
(d) provides additional resources to facilitate and operationalize the co-creation process at each stage of the NBS development, offered in the form of a Toolbox.

In order to develop a co-creation pathway and produce an innovative easy-to-use guidance for practitioners, we used a combined analysis of the literature together with various workshops and webinars with RECONECT partners on theory and practical application of the co-creation process of NBS, which relies on a participatory process. Thus, a traditional / narrative literature review was used to establish a theory and the context for a research on co-creation and its use for NBS implementation (theoretical literature review) as well as to outline methods and research design that have been used in the related field (methodological literature review), which was conducted using search words within standard literature databases (ISI WoS, SCOPUS, Google Scholar, etc.). It also based on a systematic review of large data and knowledge base of NBS that was earlier developed within the CONNECTING Nature project [22]. In particular, a methodology of such knowledge and data-base was developed with extensive NBS stakeholders’ and end-user's involvement; this experience was also reflected by development of the presented co-creation pathway. A variety of the co-creation approaches and tools developed by other NBS projects in Europe and worldwide was also considered. The RECONECT project partners were actively involved in the producing this co-creation approach. The stakeholders ranging from academia and research institutions, public authorities and private companies to media, network organizations and the individuals and communities involved in the co-creation of NBS; they are all invited to use this RECONECT co-creation/pathway /methodical approach which will guide them in the field of co-creation processes and can be flexibly adapted to different local contexts.

## Method

### RECONECT co-creation strategy: pathway and seven steps to follow

To foster a co-creative process, a variety of frameworks and tools is available [[1], [4], [8], [19], [23], [24], [25], [26]]. As they originate from different professional backgrounds (e.g. marketing, social science, food security, design research, sustainable transition, etc.), it is not evident that researchers and practitioners dealing with NBS are aware of these tools, or able to select and use them in a meaningful way. Thus, the presented RECONECT co-creation approach/pathway describes the co-creation development process and provides a step-wise guide. It combines a well-known business model with tools from design thinking that promote active participation by all relevant stakeholders. Some of the tools are common and actively used in the co-creation process of many NBS related projects. Other tools have not previously been applied to NBS; however, due to their high potential and expected applicability, they are also suggested for co-creation of NBS.

**Fig. 1:**
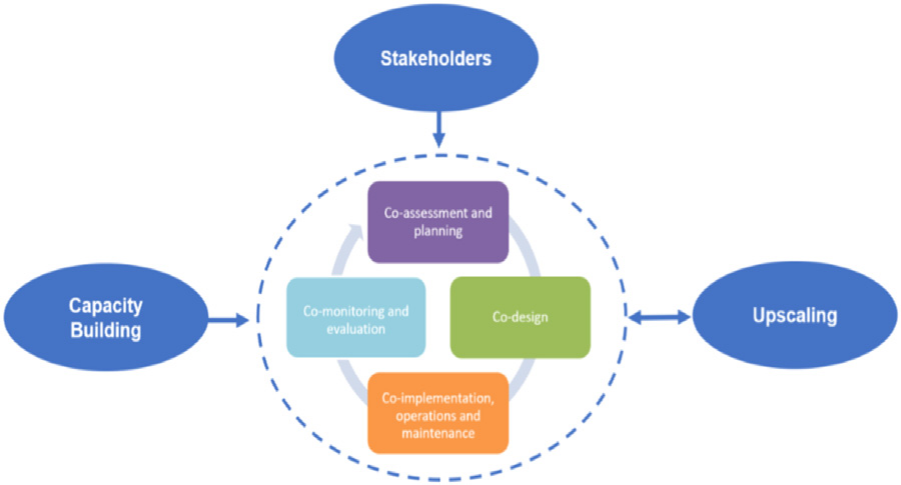
**RECONECT's Social Innovation Approach to NBS underpinned by co-creation** (“co-” in the title to each stage of NBS process presented in the circle, e.g. co-design, means involving participatory processes within the concept of co-creation) **(**Source: www.reconnect.eu).

The RECONECT social innovation approach is underpinned by co-creation processes involving researchers and various groups of stakeholders iteratively throughout the stages of: a) co-assessment and planning, b) co-design, c) co-implementation, operations and maintenance and d) co-monitoring and evaluation (Fig. 1). The prefix “co-“ used in the definition of each NBS stage presented in the circle of Fig. 1 directly relates to the concept of co-creation as “the collaborative generation of knowledge by working with a broad group of stakeholders from different sectors” [[3], [15], [27]]. The methodology developed within the RECONECT project and described in this paper provides an inclusive, participatory approach to the exploration, production and implementation of innovative solutions for hydro-meteorological risks reduction with a focus on NBS. Bearing in mind that the co-creation is an open process which runs throughout all NBS stages under continuous transformation, we can conclude that the co-creation concept can be not constant, as the process will change over time. This also implies that the concept needs to experience ongoing adaptation: changes and additional strategical elements need to be included.

**Fig. 2:**
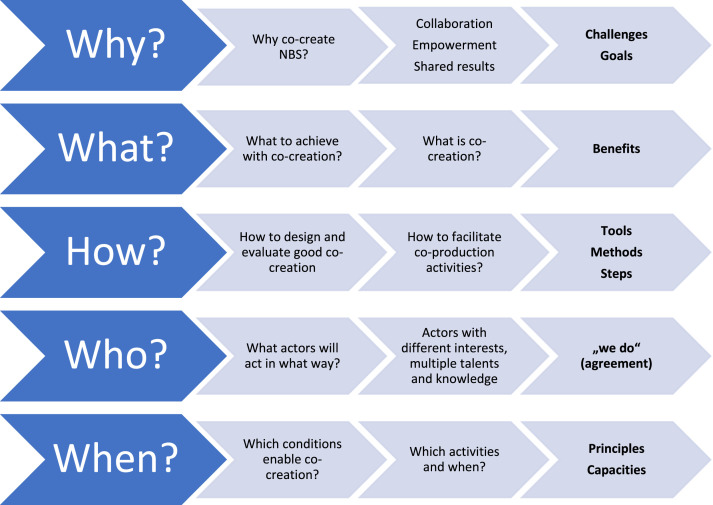
**General navigating questions in the RECONECT co-creation pathway (**Source: authors).

In developing the co-creation pathway within the RECONECT project, the following aspects/questions were considered: challenges addressed and goals to be achieved, benefits provided, steps undertaken with the appropriate methods and tools, actors involved and, finally, principles to base on as well as resources and capacities available for the process. It is inspired by Brand et al. [27] and Morello et al. [3] and designed around a set of basic questions (Fig. 2). Their goal is to support the co-creation process and relate to: (1) identifying the main objectives of co-creation (WHY?), (2) defining the main outcomes of the particular co-creation process (WHAT?), (3) explaining how, using the most relevant methods and tools, these goals and outcomes can be achieved (HOW?), and (4) helping to decide what actors should be involved in what way, and which level of involvement it requires of them (WHO?). All this occurs by considering the conditions which enable co-creation at each stage of the NBS process according to the capacities, resources and needs (WHEN?).

**Fig. 3:**
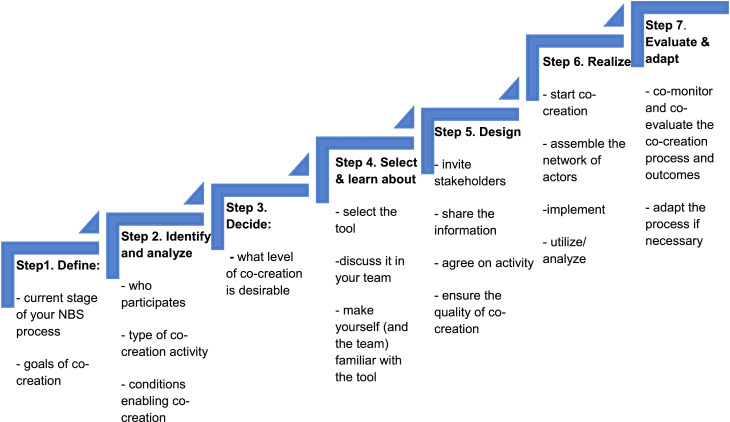
**Main steps of the RECONECT co-creation pathway (**Source: authors).

The basic questions are then translated into more procedural steps. More specifically, RECONECT's co-creation pathway consists of seven steps (see Fig. 3 and Table 1). The idea of the co-creation pathway is to support practitioners in setting-up, implementing, evaluating and adapting their co-creation process (guiding them through the process). Therefore, the foci of the RECONECT co-creation pathway are on identifying appropriate tools and methods that help to facilitate a locally adapted co-creation process.

**Table 1:** **Description of each step of the co-creation process of NBS (**Source: authors).

Step	Purpose	Guiding questions	Some options
Step 1: Define	Identifying NBS stage	At which stage of realising NBS are you?	1) co-assessment and planning, 2) co-design, 3) co-implementation, 4) co-monitoring and co-evaluation
	Defining the goal of co-creation process	What is the aim of your co-creation process?	a) exploring local context / dynamics; b) development of new visions, ways of problem framing and strategies (new business model); c) establishing new relationships, partnerships and collaborations among actors who were not in contact with each other in the past; d) stakeholders’ identification and analysis; e) foster collaboration (networks or partnerships), mobilize and empower diverse citizens for a joint action; f) stimulate learning among diverse actors; g) finding concrete solutions; h) collecting knowledge in the absence of data (data gathering); i) monitoring and evaluation of NBS impact.
Step 2: Identify and analyse	Stakeholders’ identification and analysis	Which stakeholders do you want to involve in the co-creation process?	a) public authority & political representation; b) academia & research; c) private sector organization; d) civil society organizations; e) local residents; f) media; g) several/all of these groups of stakeholders
	Identifying type of co-creation activity	What co-creation activities are you interested in applying?	a) workshops and oral communication techniques; b) fieldwork techniques; c) template and visualization techniques (templates, maps, charts, etc.)
	Analysis of conditions enabling co-creation (resources, capacities, effort)	What resources and capacities do you have for co-creation?	
		How many stakeholders will you involve?	a) small group (1–5 people); b) middle group (6–12 people); c) large group (from 13 up to 100 people and more)
		How much time will you be able to invest?	1) less time consuming (30–60 min); 2) middle time consuming (0.5–1.5 h preparation and 1–2 h activity); 3) highly time consuming (long preparation, several rounds of activities, post-event analysis)
		How experienced are you in doing co-creation?	1) low effort/level of difficulty, easy to apply; b) middle effort/level of difficulty, needs certain expertise to apply; c) high effort/level of difficulty, difficult to apply (need trained personnel)
Step 3 Decide	Level of co-creation	What level of co-creation is desirable for you?	1) Informing stakeholders about NBS (idea sharing, advertisement, educate): one-way, passive engagement; 2) consulting stakeholder(s) and informing them (surveying, interviewing, reviews, rating): two-way dialog to enhance feedback; 3) starting/supporting collaboration (delegation, consultation, partnership, engagement): initial engagement; 4) cooperating with stakeholder (partnership, engagement, delegated power, citizen control): active engagement
Step 4 Select	Selecting the most appropriate tools	What are the most appropriate tools supporting your co-creation activities?	a) select the appropriate tool(s) and look at the comparative chart to learn which efforts and resources are needed for their implementation; b) within the co-creation team it should be discussed: what parts of the tools will be beneficial to apply; c) make your project team familiar with the tool (learn how to apply, what to prepare, etc. – see tool descriptions)
Step 5 Design	Invite	How will stakeholders be invited?	1) direct (through invitations); 2) indirect (public advertisements, internet, social media, newspapers, TV, radio, etc.); 3) motivation (extrinsic motivation / financial; intrinsic motivation / social inclusion)
	Share	How will relevant information be shared with stakeholders?	a) using email or sending digital information sources; b) sending prospects, brochures, booklets, etc.; c) through direct contact (also during public events); d) using social media, etc.
	Agree	How to ensure a mutual understanding?	1) memorandum of agreement, contracts, other official documents, 2) informal agreement but indicating the activity conditions & tasks
	Ensure the quality of co-creation	What are key principles for a good co-creation process?	1) inclusivity 2) openness 3) legitimacy 4) actionable knowledge for policy and planning 5) usable knowledge and empowerment 6) extending institutions for ensuring synergies
Step 6 Realize	Starting / continuing co-creation	What key aspects to consider during realising co-creation activities?	looking at the tool descriptions, please follow the instructions for applying the tools and finally launch innovation partnerships
		How to assemble the network of actors	- recognize the roles and responsibilities of co-creation participants
		How practically implement the co-creation activity?	1) ensure that the implementation proceeds according to the plan; 2) prepare for uncertainties in the process and be prepared for changes and adaptation; 3) the only way to learn which method or tool works best in specific situations is by testing and applying it
		How to utilize the co-creation results & experiences	1) ensure that all the parties benefit; 2) analyse & share the risks; 3) an open attitude and learning through experiments are the surest means of producing benefits; 4) ensure an open, trusting, pro-development, encouraging and creative atmosphere
Step 7 Evaluate and adapt	Co-monitor and co-evaluate the co-creation	How did we do? Can we improve?	a) Reflexive monitoring; b) Monitoring the effects of using the innovation; c) Table of indicators for evaluation of co-creation process

While *steps one to four* (of Fig. 3) are rather preparatory, *steps five and six* are concerned with the realisation process (i.e. doing co-creation with stakeholders); *step seven* is about evaluation and adaption if necessary. Each of the steps is connected with a specific intermediate goal. The single steps are interconnected and they ideally build upon each other. However, iterations between single steps and also changes of order may be necessary. Co-creation is an open process, therefore adaptation and changes should be expected and prepared for. It is also important to identify and – as early as possible – reach out to the actors/stakeholders who need to or should be involved in each co-creation step.

In the following we provide a short overview of the single steps. Each of the steps is built around a set of questions. The answers to the question will help / navigate users in identifying appropriate tools that facilitate their co-creation process. The practical application and the follow-up steps are described below. In order to support users in the co-creation process, a multi-criteria decision-making matrix was developed. It provides a step-by-step guide which will help in selecting the most appropriate co-creation strategy. Each step consists of a set of guiding questions; their answers will contribute to finding the right tools for the co-creation of NBS.

**Step 0 Stage of realising NBS project**

**At which stage of the NBS project are you?**

Each stage of the NBS process (e.g. co-assessment and planning, co-design, co-implementation, operation and maintenance, co-monitoring and co-evaluation) is connected with different co-creation activities and goals: for each NBS stage we have identified different tools and methods to achieve the co-creation goals.

The following steps (from zero to three) should ideally be taken before the co-creation process starts. They are concerned with a sound preparation of the process and support the identification of tools that help to facilitate the process.

**Step 1 Define**

**What is the aim of your co-creation process?**

Before doing anything else, you should start thinking about which goal you want to achieve with your co-creation process. There may be several goals, they can overlap and some goals might be more pressing than others which might be more relevant in the long run. For instance, exploring local context and dynamics in a collaborative way can also contribute to finding concrete solutions and establishing new relationships, partnerships and collaboration. At the same time, stimulating learning among diverse actors can foster collaboration or partnership and mobilize as well as empower diverse stakeholders and citizens for a joint action. Goals might also change as the co-creation process is evolving. However, defining the goal of the co-creation process is a relevant first step. Here are several example goals as an aid to defining the goals which the co-creation should follow:

• explore local context / dynamics;
• develop new visions, ways of problem framing and strategies (incl. new business model);
• establish new relationships, partnerships and collaborations among actors who were not in contact with each other in the past;
• identify stakeholders’ and analyse their relationships;
• foster collaboration (networks or partnerships), mobilize and empower diverse citizens for a joint action;
• stimulate learning among diverse actors;
• find concrete solutions;
• collect knowledge in the absence of data (data gathering);
• monitor and evaluate NBS impact.

It is essential to document the goals underlying your co-creation process and also to provide criteria for measuring the achievement of these goals.

**Step 2 Identify and analyse**

**Which stakeholders do you want to involve in the co-creation process?**

It is important to identify with whom you are going to co-create, and to try to understand how they might influence the co-creation process: in particular, what their interests are (see Table 2) and whether they would be willing and able to contribute. There are a number of tools which can be applied only for co-creation processes with very specific groups of stakeholders. Other tools, however, also help to identify the needs and capacities of the stakeholders better, in order to enable better collaboration.

**Table 2:** **Main groups of stakeholders and the reasons for their engagement in the co-creation of NBS** (Source: authors).

Stakeholder group	Reasons for its engagement	Type of innovation created and the role	Needs and requirements	Expectation from the NBS project
Academia & research (centres) (knowledge-based organizations)	Developing synergies Experimental data on the area of interest Collaborate in developing technical standards and guidelines	Technical innovation: - actor fundamental in knowledge production; - contributor to innovation thanks to crucial role that knowledge has gained in development processes	Exchange of scientific knowledge Knowledge transfer	New scientific knowledge on environment New knowledge on socio-economic issues and participatory methods Scientific publications Broaden networks Follow-up research grants
Public authority & political representation	National, EU and global environmental strategies / standards related to NBS Develop and enforce rules, laws and regulations Provide data, permits, authorizations, institutional support Owner and manager of area	Governance and organisational innovation: - support both industry and academia for the application of ideas to development through policies, strategies and initiatives; - cross-check that the new ideas create value for society	Opportunity to develop better policies and management intervention Performance-based evidences (co-benefits for social and political purposes)	Innovative solutions and guidelines to support environmental policies and management strategies Public awareness
Private sector organization	Strong actor who leads technological and organizational innovation; generates, produces and distributes products and services	Market innovation: - strong actor that leads technological and organizational innovation; - has the role of generating, producing and distributing products and services	Opportunity to co-finance NBS project and support in the promotion of NBS, making it marketable	New green business opportunities, shared ownership, access to better technologies as well as opportunities to influence research
Civil society organizations	Joint effort toward cooperation, dissemination and exploitation Foster participation, actions and promote participatory approach Collaborate in operationalizing NBS and provide support to data collection activities Provide publicity	Social innovation: - innovation users who provide knowledge about their needs, experiences and expectations; - directly affected by any changes made by NBS and thus can provide first-hand information on challenges & enablers	Enhancing the quality of the area and landscape Public awareness and citizen participation	Evidence-based data on the efficacy of the NBS project Citizen participation approaches for the protection of the area from the societal challenges that NBS is addressing New management practices for the local area.
Media	Promoting NBS and disseminating results	Cross-sectoral innovation: - innovation users who promote the NBS and disseminate results	Awareness raising about NBS	Involvement in the actual real-life research and generating public interest

**What co-creation activities are you interested in applying?**

After identifying the stakeholders to be involved, you should become more specific about the kind of co-creation activity you would like to pursue. Various techniques may be applicable [28]:

(a) **Templates and visualisation techniques** are helpful in order to involve people with different backgrounds and education types and to achieve knowledge systematisation and consensus. Matrices are logically organised views of various information and ideas for comparison or ranking according to importance. Maps and charts are simplified representations of reality; they are particularly useful at the beginning stages of a participatory process. Flowcharts are diagrams which illustrate the relationships between different elements, often with cause-and-effect relationships or sequences of events. Timelines show the presence, absence and intensity of certain phenomena over time.
(b) **Workshops and oral communication techniques** are helpful in order to collect a diverse set of information and to unravel different views and perspectives. They focus on information to be triangulated from distinct points of view of stakeholders (selection of key respondents, focus groups) as well as from their views on a specific set of problems (semi-structured interviews).
(c) **Field work techniques** help to gather information in the field, from a group perspective and utilising visualization techniques to analyse the data.

**What resources and capacities do you have for co-creation?**

In a next step, some relevant contextual factors need to be considered. We suggest that, in order to select the right tool for the particular co-creation goal, it is advisable to assess the resources, capacities and efforts necessary in order to set up the process (Table 3). This includes:

• **With how many stakeholders will you co-create?** Define the size of the group of stakeholders to be involved: (i) small group: 1–5 people; ii) middle group: 6 to 12 people; iii) large group: from 13 up to 100 people and more;
• **How much time will you be able to invest?** Define how much time you will be able to invest in the co-creation process. We suggest distinguishing the activities into three different clusters: i) less time consuming: 30–60 min; ii) middle time consuming: 0.5–1.5 h preparation and 1–2 h activity; iii) highly time consuming: long preparation, several rounds of activities, post-event analysis;
• **How experienced are you in doing co-creation?** Define your level of expertise in doing co-creation activities and conducting, for instance, participatory workshops. We differentiate three levels of expertise: i) tools that are relatively easy to apply; ii) tools that need certain expertise to apply; iii) tools that require profound expertise/experience.

**Step 3 Decide on level of co-creation**

**Table 3:** Conditions enabling co-creation: resources, capacities and effort required, in order to select the most appropriate tool (Source: authors).

Size of stakeholder group	Time frame	Level of expertise / effort needed
o Small group (1–5 people)	o Less time consuming (30–60 min)	o Relatively easy to apply
o Middle group (from 6 up to 12 people)	o Middle time consuming (0.5–1.5 h preparation and 1–2 h activity)	o Needs certain expertise to apply
o Large group (from 13 up to 100 people and more)	o Highly time consuming (long preparation, several rounds of activities, post-event analysis)	o Needs expertise and experience

**What level of co-creation is desirable for you?**

**Fig. 4:**
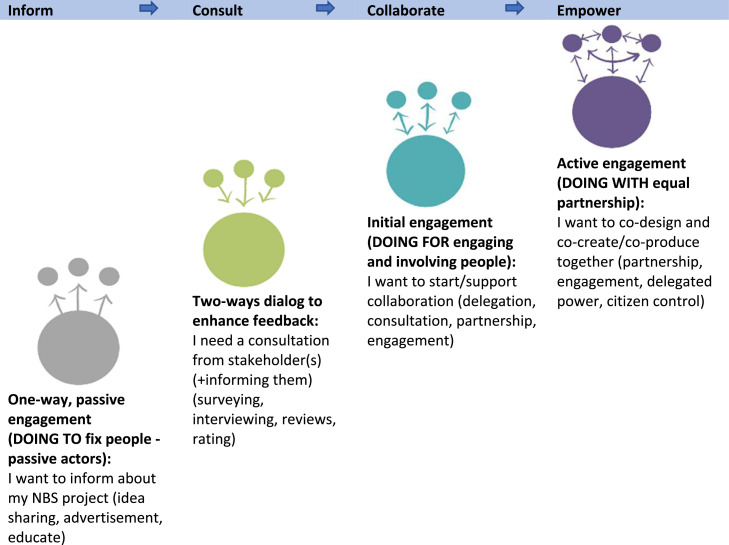
**Levels of co-creation according to stakeholders’ involvement in NBS (Source: authors)**.

Each level of co-creation aims at certain engagement activities expanding from (Fig. 4):

• Informing stakeholders (one-way passive engagement, when the co-creator aims to inform about the project),
• Consulting (based on the two-way dialogue aiming to get a consultation in form of interviewing, surveying, etc.),
• Collaborating (initial engagement where we involve people in the co-creation through the establishing of partnerships etc.),
• Empowering (active engagement where stakeholders are the main actors and equal project partners).

**Step 4 Select appropriate tool(s)**

**What are the most appropriate tools supporting your co-creation activities?**

In this step, you choose tool(s) that are most appropriate for facilitating your co-creation process. Specific co-creation tools facilitate each step of the process towards desired goals. The choice of tools depends on the answers you gave to steps zero to three, more specifically on the goals of the co-creation process and the specific co-creation stage (which were defined in the step 1), on the type of actors involved (step 2) and type of the desirable co-creation activity and the level of co-creation (step 3). Moreover, the process of selecting the co-creation tools should consider the materials, time, skills (effort), cost and other requirements needed in order to apply the particular tool. For this purpose, we have developed a decision-making matrix in the form of a comparative chart of all tools in order to support the selection process (Table 4). All 88 tools are provided in the Manual for practitioners (see [9]) in form of factsheets of tools and listed below in Table 5, Table 6, Table 7, Table 8, Table 9.

**Step 5 Design**

**Table 4:** **Decision-making matrix – comparative chart of all tools provided in detail in the RECONECT Manual for practitioners**[9]**to support the selection process of Step 4** (Steps 0-3 are indicated in the table and described earlier) (Source: authors)

**Table 5:** **Co-creation approach and related tools for co-assessment and planning stage (Source: authors)**.

What	Why	How
Stakeholder involvement in data collection on hazards, exposures and vulnerabilities	to validate and adapt expert estimates and models of risk; to get valuable insights from stakeholders on problem framing	Visualization techniques to solicit stakeholder ideas, concerns, feedback: - value proposition canvas, A/B tests to compare risk model outputs to experiences of stakeholders; - problems definition, calls for ideas and experience tour to frame the problem on the way to the right solution; - causal loop diagrams to adapt the risk assessment model to local contexts, including feedback across social, economic, ecological governance dimensions; - citizen science, fuzzy cognitive maps and PPGIS for model development and analysis; - fuzzy cognitive maps to elucidate risk perceptions to support NBS; -geoquestionnairesurvey to provide input from stakeholders on placement of NBS to mitigate risks; social media allows citizen-collected information to be time-stamped and georeferenced. Field work techniques: transect walk, auto-photography, field trips, ethnographic fieldnotes, social mapping,sketch mapping.
Communication with stakeholders	to effectively mobilize participants; to provide feedback on collected data	Workshop and communication techniques to collect risk perceptions and conduct vulnerability assessment: Delphi methods /surveys, fuzzy cognitive maps, focus groups, expert interview,vision development workshop, road mapping workshop, ambition setting workshop,world café, future searching conference. Workshop and communication techniques to find the relations between the stakeholders, mobilize them, establish new and foster existing partnerships: people and connection maps, team canvas, who inspire us, building partnership map,service blueprint
Setting up the system boundaries through baseline assessments	to collect data on current working routines, institutional structures, needs related to planning, design, constructing & maintaining NBS	Visualization techniques: framing the project board, innovation flowchart, designing the challenge, experiencetour, call for ideas, user personas, actors map, stakeholder visualization, stakeholderCV tool, people shadowing Workshop and communication techniques: focus group, expert interview, vision development workshop Field work: transect walk, auto-photography, field trips, ethnographicfieldnotes, social mapping.
Selecting the criteria to address the issues of Water, Nature and People indicators	to adjust criteria to ensure local relevance of NBS to environmental, health, economic, socio-political, technical issues.	Visualization techniques: multi-criteria decision analysis, scoring and rating, multiple perspectives wheel,scaling plan Workshop and communication techniques: Delphi survey/methods/techniques, focus group, expert interview, visiondevelopment workshop, world café, dotmocracy
Comparing selected NBS to other types of measures	to analyse all pros and contras of selected NBS and their co-benefits	Using a combination of cost assessments, qualitative and quantitative assessment of co-benefits, and evidence synthesis of existing literature; co-creation tools: scenario comparison, A/B tests (split tests), SWOT Analysis, expert interview,thinking hats, head & heart & hands, 5 whys, dotmocracy, world café
Selection of stake-holders participating in co-creation	to identify the interested stakeholders and ensure their engagement	Can be derived through the stakeholder mapping and analysis; Visualization techniques: stakeholder visualization, user personas, actor maps/stakeholder mapping, stakeholderCV tool; Workshop and communication techniques: focus group, expert interview,world café, team canvas, who inspire us, building partnership map, service blueprint

**Table 6:** **Toolbox for co-assessment and planning of NBS: aims, outcomes, actions/steps, actors to be involved and related tools** (Source: authors, inspired by [[3], [15], [27]]).

Phase	Co-assessment and planning		
Main aims and actions/steps:	1) defining the current and future problem, challenges (Who and what is at risk?); 2) finding possible solutions in relation to NBSs opportunities – e.g. motivation for NBS and its main objectives (preliminary research) (What is the purpose of NBS?) 3) understanding potential for NBS: defining operational technical skills to design the project (What are the benefits and costs?) 4) clarifying the role and mission of different stakeholders involved in NBS (Who is on board, with whom and why?) 5) Identification of the NBS project – achieving its sound vision (What are feasible & acceptable NBS types?)		
Why participatory tools should be applied	• to establish a sound vision of the NBS project and the alignment of partners • to improve the overall involvement of all relevant stakeholders in the process (increasing awareness about the NBS project)		
Outcomes:	1) report including the identification of the NBS project and its relation to the overall local strategy; 2) list of related stakeholders and their roles in the project; 3) prototype of local communication, outlining the strategy for gaining stakeholders’ support and engagement for NBS, set-up activities		
Actors involved:	Project partners: only internal local working team and sporadically, experts and decision-making board from public authorities		
Important notes:	Since this step contributes to the establishing the partnership: • make sure to invite all stakeholders and align with them; • make sure to provide all needed materials for tools applied for this stage		
Suggested tools:	a) for identifying the challenges and goals of the NBS project within the local context and strategy:	• framing the project board • innovation flowchart • designing the challenge • experience tour • problems definitions • value proposition canvas	
	b) to involve and engage stakeholders	• user personas • actors’ map/stakeholder mapping • people and connections map • stakeholder CV tool • people shadowing	• expert interview • stakeholder visualization • team canvas • service blueprint • who inspire us? • building partnership map
	c) to discover valuable insights and generate innovative NBS strategy	• brainstorming/ brainwriting • walls of ideas • fast idea generating • idea dashboard • idea canvas • co-co toolkit • idea rating	• creative workshop • expert interview • 5 whys • thinking hats • head & heart & hands • action Planning • SWOT analysis
	d) to collect data on hazards, exposures and vulnerabilities	• citizen science methods (incl. digital photos and videos from the public); • fuzzy cognitive maps • participatory mapping, PPGIS, geo-questionnaire • participant observation • transect walks • focus groups

**Table 7:** **Toolbox for co-design stage of NBS: aims, outcomes, actions/steps, actors to be involved and related tools** (Source: authors, inspired by [[3], [15], [27]]).

Phase	Co-design		
Main aims:	the involvement of stakeholders in designing or rethinking an output (in our case: a nature-based solution) (WHY?), through direct collaboration with the design team during the development process (HOW?)		
Actions / Steps	• Share and update the NBS focus through citizens’ contribution • Co-design specific NBS alternative scenarios • Evaluate the co-designed scenarios and converge on the solution. • Disseminate the NBS co-design status • Communicate and update specific NBS through various dissemination channels • Evaluate the NBS design dissemination modality • Assess and test the final co-design scheme		
Why participatory tools should be applied	This is the activity that mostly involves local stakeholders and citizens in a participatory process, and thus requires a highly inclusive approach and communication effort.		
Outcomes:	• Co-design project proposal • Dissemination plan about co-design communication (including particular actions and events related to co-design) • Final co-designed proposal		
Actors involved:	A variety of people that best represents the local context: respect gender equality, avoid discrimination, cover different ages and professional skills. It is crucial to get the same people during all the events in order to consolidate the narrative of the whole process. In the current situation, e-participants form a valuable group and should be considered in the same way as physical participants.		
Important notes:	• The richer the composition, the higher the success of the activity will be. • Build loyalty to the challenge and make sure people come back; small rewarding strategies and personal recalls can help.		
Suggested tools:	a) co-design workshops	• vision development workshop • creative workshop	• ambition setting workshop • road mapping workshop
	b) tools to design action plans (strategy) to achieve long-term aims	• business model canvas • co-implementation scheme/plan • evidence planning • system map	• motivation matrix • causes diagram • theory of change • casual loop diagram • scenario planning • idea canvas tool card
	c) tools to unleash creativity, discover valuable insights and generate innovative NBS	• brainstorming / brainwriting • idea dashboard • fast idea generating • idea canvas • walls of ideas • thinking hats	• 5 whys • multiple perspectives wheel • story world • co-co toolkit • head & heart & hands
	d) tools to select, support decision and evaluate users’ reaction	• dotmocracy • transformative impact • logic model • I like, I wish, what if • multi-criteria decision analysis	• scaling plan • heuristic evaluation • scoring and rating • Delphi survey / methods / techniques

**Table 8:** **Toolbox for co-implementation, operations and maintenance of NBS: aims, outcomes, actions/steps, actors to be involved and related tools** (Source: authors, inspired by [[3], [15], [27]]).

Phase	Co-implementation, operations and maintenance	
Main aims:	• through working with people and partners, to put the solution into action; • the tangible interventions serve as a ‘test’ environment to make NBS marketable and sustainable; • participation in implementation, putting into practice activities	
Actions / Steps	• co-implement the joint NBS project (develop place-specific co-implementation plans outlining arrangements aspects and commitments) • verify the NBS co-implemented action in place (assess the co-implementation scheme previously planned)	
Why participatory tools should be applied	They are a crucial for assessing the scopes of the NBS project and testing ambitious partnerships and social innovation solutions in the co-implementation phase. The best suitable alternative design project selected in the previous co-design phase is ready for construction and looking for partners to prototype the NBS or directly implement it. Hence, it is fundamental to involve local business and companies that can deliver the solutions, also with original sponsorship schemes. For instance, during the technical implementation phase, some new topics might emerge and have to be considered by the NBS site responsible person. In this step, a final reality check of the solution is required	
Outcomes:	Primary implementation scheme; revised Implementation scheme	
Actors involved:	All local stakeholders, including sponsors, suppliers and individual citizens who might contribute to the implementation, operation and maintenance phase, depending on the possibilities that the construction offers.	
Suggested tools:	Tools to test and validate the developed NBS:	• prototype testing plan • live prototyping • usability testing • assumption mapping • blink testing • A/B tests • story boarding • improvement triggers • service Blueprint
	Tools for experimenting:	• planning support system • I like – I wish – what if • cool wall
	Tools to support decision:	• cool wall • dotmocracy • assumption mapping • planning support system
	Tools to co-produce, co-implement and co-maintain:	• co-implementation scheme/plan • role script • actors map
	Tools for co-governance:	• actors map • role script

**Table 9:** **Toolbox for co-monitoring and co-evaluation of NBS: aims, outcomes, actions/steps, actors to be involved and related tools** (Source: authors, inspired by [[3], [15], [27]]).

Phase	Co-monitoring and co-evaluation
Main aims:	1) to evaluate the implemented NBS and monitor the durability and quality of the interventions; 2) to reflect on co-monitoring and co-evaluation of the co-creation process and its activities
Why participatory tools should be applied	• it requires a strong involvement of stakeholders who can support by measuring the success of the complete process. Indeed, this is a crucial point on the pathway, where a strong effort to sustain all the process is required. This step requires original solutions, cohesion and constancy. • diverse stakeholders (especially the end-users / beneficiaries of the NBS) will contribute to assessing the impact of the interventions and success or failure of processes; • citizen science approaches can be used to collect data in order to monitor and evaluate the implementation progress of NBS development.
Actions/steps:	• Validation of NBS in place • Verification of NBS co-benefits in place • Evaluation of co-creation process
Outcomes:	Reports on monitoring activities; evaluation protocols
Actors involved:	All local stakeholders, project team, evaluation team
Suggested tools:	• logical framework analysis • focus group discussions • social mapping • PPGIS • geo-questionnaire • beneficiary assessment • participant observation • transect walk • scenario planning • participatory mapping • scenario comparison (before / after)

**How will stakeholders be invited?**

Invite your stakeholders and suggest/agree on how they will be involved/engaged. There are various ways of inviting stakeholders to the co-creation process:

• direct (through invitations),
• indirect (public advertisements, internet, social media, newspapers, TV, radio, etc.),
• motivation (extrinsic motivation, e.g. financial; intrinsic motivation, e.g. social inclusion).

Decide what is desirable and doable at the current stage of your NBS process and according to your particular co-creation goal.

**How will relevant information be shared with stakeholders?**

Share with stakeholders the essential information in advance in order to prepare the co-creation process. The process of information sharing with the relevant stakeholders can be organized in various ways: using email or sending digital information sources; sending prospects, brochures, booklets, etc.; direct contact (also during public events); using social media, etc. There is a number of collaborative software which can support in this regard (e.g. https://en.wikipedia.org/wiki/List_of_collaborative_software).

**How to ensure a mutual understanding?**

Agree with stakeholders about the type of co-creation activity that they will participate in. In order to ensure that you agreed on the preferable type of co-creation activity, various instruments can be used, such as memorandum of agreement, contracts, other official documents, informal agreement but indicating the activity conditions & tasks.

**Step 6 Realize**

**What are key aspects to consider when realising co-creation activities?**

After looking at the tool description, follow the instructions on how you can apply the tool and finally launch innovation partnerships. Next, you assemble the network of actors for co-creation where you recognize the roles and responsibilities of co-creation participants identified in the previous steps. Only then can you start the practical implementation of co-creation by:

• ensuring that the implementation proceeds according to the plan;
• checking that you are able to tolerate uncertainty and are prepared for changes;
• considering that the only way to learn which method or tool works the best in various situations and between various actors is by testing.

Once the co-creation is started, you have to think about the utilization of the co-creation results and experiences. Here, it is important to be sure that all the actors involved in the process benefit. Do not forget to think about the risks and share the results of your risk analysis. At this step, an open attitude and learning through experiments are the surest means of producing benefits. Ensure an open, trusting, pro-development, encouraging and creative atmosphere.

**Step 7 Evaluate and adapt**

**How did we do? Can we improve?**

By definition, co-creation is an open process which evolves over time as learning progresses. Each co-creation process “goes with the flow” of the participants’ ideas and needs [15]. This requires continuous monitoring, reflexivity and evaluation. Reflexivity helps to identify lessons learned and to adapt the process in the light of changing objectives. Therefore, those involved in co-creation should ask many questions about the process along the way, for instance:

• which goals does the process aim to achieve?
• is the process on the way to achieving these, or do we need adaptations/modifications?
• how can we improve based on the lessons learnt?
• what impact has the process had?

One of the methods for achieving this reflexivity and support by answering the above questions is “Reflexive monitoring in action” (for more details see [[6], [15]]). Another approach to evaluate the impact of co-creation is to compare the situation before and after. A methodical approach, together with guidelines a set of indicators, to monitor and evaluate the impact of co-creation is currently under development and will be provided soon in the separate document.

### Toolboxes to support the co-creation process at every NBS stage

This section provides toolboxes to all four stages/phases of NBS process: 1) co assessment and planning, 2) co-design, 3) co-implementation, operations and maintenance, 4) co-monitoring and evaluation). Every toolbox consists of many participatory methods and tools and explains for what particular goal, outcome and how they can be put into practice.

**Toolbox for the co-assessment and planning phase of NBS**

The co-assessment and planning stage of the NBS process is aimed at analysing and understanding the existing situation; in particular, the following questions are of relevance:

• who and what is at risk/face the challenge?
• what are feasible and acceptable types of NBS?
• what are the benefits and costs?
• who and how to engage in co-creation?

In order to respond to these questions, the work should focus on the following steps: a) identify the proposed NBS project within the local context and strategy; b) map and engage stakeholders; c) launch the innovation partnerships within the NBS project. More specifically, it will include assessment of risks in response to the questions:

ü How are stakeholders exposed to hydro-meteorological hazards?
ü What are their vulnerabilities, expectations, needs and capacities to implement NBS?
ü Which other risk mitigation options can be defined?

Based on these assessments, the applicable types of NBS and their feasibility should be determined. Finally, the assessment of different types of NBS should be carried out in relation to benefits, co-benefits, and cost-assessments that reflect Key Performance Indicators. During this stage, co-creation activities can ensure that contextual factors are considered in risk assessments, such as local cultural, social or biophysical factors.

This stage includes the co-creation activities employed to assess hydro-meteorological hazards and risks caused by them, feasibility assessments to explore the best options according to a range of criteria and carried out through a co-creation approach, as well as assessments of benefits, co-benefits and costs of NBS.

### Risk assessment of hydro-meteorological hazards

Defined as a process which facilitates exchange of information between scientists, decision-makers and citizens, co-creation is increasingly important in order to respond to hydro-meteorological hazards. Especially important is a stakeholder involvement that goes beyond limited consultation and results in collaboration (stakeholder engagement), which offers a number of benefits [27], particularly the sharing of responsibilities in flood risk management. One such method refers to the *citizen science* approach, which is particularly important in order to address scarcity of data needed for risk assessment across temporal or spatial dimensions.

A participatory approach is important for assessment of vulnerability, risk perceptions, and local priorities; it provides critical information to enable NBS selection, for example through methods such as fuzzy cognitive maps, which have been used to elucidate risk perceptions to support NBS [29]. Participatory modelling experiments play a role for hydro-meteorological risk model development related to computer simulations helping to generate new information about flood risks drawing on local knowledge, contributing towards more active stakeholder participation and a ‘redistribution’ of expertise [[30], [31], [32], [33]]. In this regard, equitable ways of working and ongoing communication are critical in such processes, which may require a greater investment of resources but can result in greater societal connectivity to hydro-meteorological hazards and their impacts, various opinions and perspectives to be reflected upon and considered. Within RECONECT, the co-creation approach to risk assessment can be conducted and adapted to particular needs in the case study settings regarding the following aspects presented in Table 5, using the tools suggested in Table 6.

### Feasibility assessment

Feasibility assessment of NBS aims to define the potential measures that are deemed effective, sustainable and acceptable in relation to the location. This process also considers a range of environmental, social, economic, technical and human well-being criteria. The feasibility assessment is applied by using local knowledge, scientific knowledge and the appropriate technological means. For the purpose of mainstreaming a methodology that can be generic enough to allow for cross-case comparison of feasibilities of different NBS and which can also capture site-specific conditions and allow for the contextualization of criteria evaluated, the RECONECT approach will allow for continuous involvement from stakeholders from early stages of the feasibility assessment and selection process. The approach to feasibility assessment combines both preliminary selection of measures and analysis of stakeholders’ preferences.

### Preliminary selection of measures

RECONECT is developing the knowledge base of NBS measures for hydro-meteorological risk reduction, which will sit on a web-based platform and be structurally analysed using the RECONECT scaling strategy (see www.reconect.eu). Stakeholders and decision makers will be able use this platform to select feasible measures according to their potential to achieve desired benefits and co-benefits. The measures for selection of suitable NBS for a specific area will be preliminarily based on: 1) the conditions of the affected area (e.g. urban, river basin, coastal or mountainous); 2) the type of hazard (e.g. floods, droughts, storm surges and landslides); 3) land characteristics (e.g. land use type, soil type and depth, topography, available space, drainage area and infiltration rate) (see www.reconect.eu).

### Analysis of stakeholders’ preferences

Collaboration with different local stakeholders can help to explore how acceptable, sustainable, and effective the preliminary selected measures are in relation to the location and how they reflect (consider) a range of environmental, social, economic, technical and health criteria. For this purpose, a Multi-Criteria Analysis (MCA) can be used to evaluate the feasibility of different NBS. Included in the MCA are decision models which contain options that need to be ranked or scored by stakeholders through a set of criteria, and a set of performance measures, which will be the raw scores for each decision option against each criterion. The criteria in this analysis include selecting indicators from different categories (e.g. related to water, nature and people impact/characteristics).

### Economic benefits, co-benefits and cost-assessments

NBS are all different in shape, size, service provided, local conditions, design, and construction and maintenance cost. They can provide a range of benefits and co-benefits spanning across the three challenge areas: Water, Nature and People. Acknowledging this variability is an important pre-requisite when it comes to assessing their economic and business impacts. Current research available on this topic, in particular on business models and governance schemes to distribute value, was conducted and further developed by CONNECTING Nature [10,15], NATURVATIONS and other projects. Considering their main perspectives and ideas, RECONECT applies two approaches that could be used to identify NBS impacts:

• the ‘Ecosystem Services’ approach, which puts more focus on the Social component of NBS (human health and well-being);
• the ‘Total Economic Valuation’ approach, which is a more global and integrated method that can spot impacts on the Nature and Environment aspects of the NBS.

NBS impacts identification framework and Total Economic Valuation framework, helping to asses all the values and especially economic impacts generated by NBS, can be found in Handbook for Practitioners on evaluating the impact of NBS [34]. Once impacts are identified, several methods exist to quantify them economically, e.g.: a) the Market Valuation Approach, b) the Revealed Preference Approach and the c) Stated Preferences Approach. These valuations need good local knowledge (culture, standard of life, real estate, habits of consumptions of locals, etc.) and good modelling tools (multiple parameters and data that need to be integrated). It is particularly complicated to estimate the monetary value of a whole NBS with this approach (especially in case of a large-scale NBS). This approach is more relevant for showing a trend or habits than for obtaining accurate assessments of NBS. Stated Preference Approach can be perceived as a simulated valuation method, which can be applied using questionnaire survey (e.g. Contingent Valuation Method) where respondents can be asked to express their willingness to increase the level of water quality in a stream, lake or rivers so that they might enjoy activities like swimming, boating, or fishing. Also, approaches such as choice modelling or group evaluation can help through choice experiments, contingent ranking, contingent rating and pair comparison, where a participatory approach can also contribute well.

Because of the diversity of variables and the difficulty of capturing the value of ecosystems, an economic assessment of NBS is complex; hence the importance of stakeholder engagement from the start. In a co-creation approach, stakeholders would be part of the discussion in order to expose the situation of their case (local needs, expectations, economic context, etc.), and, together with experts and researchers, find the best methods to assess the economic value of NBS.

Elements necessary to engage in a co-creation process for valorising NBS include:

• Clarity of the challenges addressed, type and location where the NBS would be established (geography, policy strategies in the NBS scope, etc.)
• List and type of existing or potentially new stakeholders
• Values and services provided by NBS
• Impacts on stakeholders (people), water and nature.

**Toolbox for co-design of NBS**

The co-creation process within the co-design stage of NBS addresses:

• which parts of the land will need to be reshaped?
• where will particular parts of the NBS project be located?
• how to validate and adapt expert estimates and models of risk?
• what co-design aspects should be considered? (e.g. landscape approach, designing with multiple objectives and designing with people)

At this stage of the NBS process, an analysis of different NBS design configurations and discussions with local stakeholders on how this design meets stakeholders’ needs and use will be done. In particular, design configurations such as the following can be discussed: which parts of the land will need to be reshaped, where will particular parts of NBS project be located, etc. This also includes co-design of hydro-meteorological risk models. Local stakeholder knowledge plays an important role in validating and adapting expert estimates and models of risk. Tools such as causal loop diagrams can be used to adapt the risk assessment model to local contexts, including feedback loops across social, economic, ecological and governance dimensions.

Furthermore, various different visualizations can be used to solicit stakeholder ideas, concerns, and feedback. For instance, participatory exercises can be used to compare the risk model outputs (e.g. flood outlines) with experiences of local stakeholders. Stakeholders can provide input on placement of NBS to mitigate risks using PPGIS for model development (Table 7).

In order to ensure that the implemented NBS provides as much benefit as possible (environmental, social, economic etc.), the following co-design aspects should be considered [10]:

• *Aesthetics* – NBS need to be aesthetically appealing to citizens
• *Novelty* – NBS creates new green commons
• *Trust* – experimenting with NBS is based on trust in the local government and in the experimentation process itself
• *Diversity* and learning from social innovation are the key of co-creation of NBS
• NBS is based on *collaborative governance*
• *Inclusivity* – an inclusive narrative of mission for NBS can enable integration to many local agendas
• *Replicability* – the design of the NBS should enable learning and replication in the long term.

Design of NBS requires a holistic perspective that considers the sustainability of the solution beyond its own system, so that it incorporates the social, physical and ecological dimensions of the surroundings.

Xing et al. [35] proposed that the co-design principles of each NBS should be based on:

• *landscape approach* – e.g. if working with water, then it is important to work with the whole watershed, not just the part of the waterbody that may be the most degraded site or that might present the highest frequency of floods. This is due to the fact that activities upstream can have adverse effects downstream. Particularly, land use such as agriculture could increase runoff volumes, stream downcutting and bank erosion, as well as pollutant loading. Urbanization and infrastructure development could exert pressure on existing water systems or affect water flows. A localized solution may not be able to change the entire watershed, but it can be designed to accommodate watershed effects better. At the same time, often some NBS benefits are not generated on-site, but spill over into many places inside and outside the area where the NBS is implemented, beyond the administrative limits [12];
• *designing with multiple objectives* – design options of NBS should be based on multifunctionality in order to achieve multifunctional land use with multiple objectives that can combine a variety of benefits and co-benefits. Application of this concept requires a change of thinking in terms of not only traditional practices, which place a greater focus on grey infrastructure, but also the traditional landscape planning practice, and much more on integration between the two, resulting in a multifunctional design approach;
• *designing with people* exactly relates to fostering community participation [[27], [36]] as well as embedding NBS into government regulations and local planning strategies. Community initiatives are participatory processes, which should be based on the transparent communication of potential actions; this assists in the identification and promotion of community initiatives as a base for NBS. Apart from community initiatives, which are important for creating bottom-up processes in landscape planning, government policies playing a significant role in the maintenance and operations of NBS as well as ownership and delegation of responsibilities together with the financing are aspects which need to be incorporated in the design of the NBS;
• *designing for function not for form* requires awareness at the design stage that not all NBS become functional overnight. For example, it might take several years for wetlands to start retaining nutrients or create the optimal conditions for enhancing wildlife. In general, not over-managing the NBS but allowing it time to develop, considering the principle of mimicking natural processes, is a key attitude for success.

Design of a RECONECT project incorporate a three-dimensional approach of time, space, and frequency, which is necessary in order to assess the drivers of risks, and to identify whether the risk is due to the magnitude of the hazard, the vulnerability of the case or the combined exposure of these. At the same time, each NBS serves many purposes beyond hydrological risk reduction, with some benefits being delivered on a daily basis whilst others are only evident during an extreme event. Finally, the space used for NBS is also important in the assessment of various services and multiple benefits. The potential design options of NBS should consider different requirements across the water, nature and people dimensions, which can be analysed and assessed using the related groups of indicators provided in the previous reports of RECONECT.

Even though collaborative approaches to design of NBS are an emerging trend, they are still not yet mainstream. However, there is an increasing number of reports and publications offering guidance on how to co-design NBS successfully [6].

**Toolbox for co-implementation, operations and maintenance of NBS**

This stage includes provision and construction of the co-designed NBS solution. Co-creation at this stage means working together with stakeholders (including local people) and partners to put the NBS into action and verify these actions.

Co-implementing the NBS solutions means involving people and stakeholders, especially local ones, in the provision and construction of the co-designed solution. Feeling part of the implementation phase is fundamental in order to motivate the stakeholders to care for the solution when applied. In other words, co-implementation means working together with stakeholders (including local people) and partners to put the NBS into action; the tangible interventions serve as a ‘test’ environment to make NBS marketable and sustainable. It consists of the following steps:

• Co-implement the NBS (e.g. develop co-implementation plans/scheme outlining arrangements aspects and commitments)
• Verify the co-implemented action in place (e.g. assess the co-implementation scheme previously planned).

It is important to define a Local Monitoring & Evaluation Team before starting this phase with citizens. The co-creation activities and tools related to co-implementation, operations and maintenance are presented in Table 8.

**Toolbox for co-monitoring and evaluation of NBS**

The co-monitoring and co-evaluation stage addresses the following questions:

• how effective is each co-implemented NBS in achieving desired benefits and co-benefits?
• what works, what does not work and why?
• how successful was the co-creation process and what are the lessons to be learnt?

This is the final stage of the co-creation process; it aims to compile information from implemented interventions in order to generate insights into how effective each co-implemented NBS is in achieving desired benefits and co-benefits, together with an analysis of what works, what does not work and why.

Co-monitoring targets the NBS performance characteristics as well as their adaptability in view of the proposed and unexpected changes and development scenarios. Before starting the co-monitoring activities, it is important to develop a comprehensive monitoring plan using a participatory approach. This monitoring plan should provide clear aims and objectives, identifying relevant indicators, target values and monitoring program duration as well as roles and responsibilities of the stakeholders involved. The tools and methodologies that will guide the co-monitoring and co-evaluation plan include citizen science, monitoring indicators and feasibility, tracking policy and planning processes, together with the use of web-based tools to support the process.

Co-monitoring and co-evaluation help to evaluate the impact of implemented NBS and monitor the durability and quality of the interventions. It will contribute to assessing the impact of the interventions and success or failure of processes.

Local platforms can be used to collect data in order to evaluate the implementation progress from a local NBS development perspective. This phase must be considered as a co-creation activity in all aspects because it requires a strong involvement of stakeholders; here is where the success of all the process is measured. Indeed, this is a crucial point on the pathway, where a strong effort to sustain all the process is required. It also refers to exploring and finding solutions for replicating successful stories in other NBS. Involved stakeholders should be the promoters for replication and further development of the implemented solutions. Solutions are both tangible products and innovative procedures to diffuse the application of NBS in cities and regions. It consists of the following steps:

• Validate NBSs in place
• Verify co-benefits of NBSs in place
• Sustain action for replication
• Co-develop the action

Co-evaluation facilitates understanding changes to mobility patterns and behaviours within neighbourhoods and the way in which they happen. It deals with impacts (what / how much has changed) and processes (what has led to that change, what has been done, what barriers and drivers affected the process, etc.). As the prefix “co-” implies, co-evaluation is performed jointly, in a way which is inclusive of the stakeholders participating in co-creation.

Co-evaluation is therefore important to a wide variety of stakeholders:

(a) *The users of the co-creation approach (*e.g. *cities, neighbourhoods)* – to be able clearly to demonstrate and communicate the impacts of and the processes behind the implemented measures and co-creation actions
(b) *Stakeholders dealing with similar issues – o*ffers the opportunity to participating cities and take-up cities to learn from each other and exchange knowledge and good practice
(c) *Funders and policy-makers, such as city administrations and the European Commission*
(d) *Other stakeholders, such as scholars, urban planners, project developers*

There are two complementary aspects of co-evaluation:

• *impact evaluation* is used to assess how successful a measure and/or a co-creation action is in reaching its stated objectives. To this purpose, measurements before and after implementation are undertaken. The methods employed in gathering and analysing the data are mainly quantitative;
• *process evaluation* seeks to provide a qualitative understanding of the way in which the planning and implementation process was conducted; its key component is an analysis of the drivers and barriers for the success or failure of the measures and the participation process.

Three steps of co-evaluation:

(1) **Monitoring**: includes observation of impacts and processes;
(2) **Assessment**: concerned with analysing and reporting quantitative and qualitative information from monitoring in a structured way;
(3) **Evaluation**: determining the value of the outcome (whether something was worthwhile / beneficial) and learning lessons / drawing recommendations about co-creation actions and mobility measures.

**Fig. 5:**
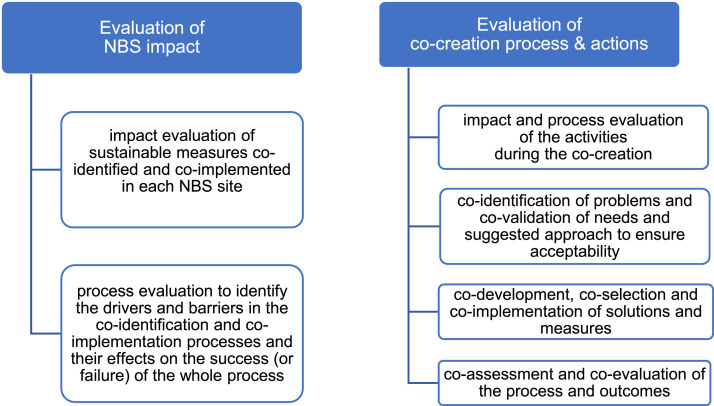
RECONECT approach to co-monitoring and co-evaluation of NBS impact and the whole co-creation process (Source: adapted from [[3], [27]], with modifications).

We suggest using a combined approach to co-evaluation (Fig. 5):

The co-creation activities and tools related to co-implementation, operations and maintenance are presented in Table 9.

## Validation

The presented co-creation pathway and step-by-step guideline were discussed and validated during the RECONECT project activities on various NBS sites. They were also analysed uand updated during a series of project-related world-café sessions, workshops and webinars in 2020–2023. The comments and feedback derived during these activities from the project partners and the experts, especially from a theoretical-practical perspective, allow the presented co-creation pathway and step-by-step guideline to be validated against the real situation and local context. The validation steps included: (a) presentation of the RECONECT co-creation pathway and supportive material (matrix of tools, toolboxes, factsheets of tools) to the project partners to identify improvement opportunities and gaps, (b) application of the RECONECT co-creation pathway and supportive material in the real-life settings/ at the NBS sites, (c) determination of approach relevance and feasibility of application to NBS sites, and (d) complete application of co-creation approach at NBS sites. Project partners feedback on/validation of co-creation pathway, matrix of tools and toolboxes relevance and applicability for the NBS sites of the RECONECT project is presented in Table 10. The research, testing, and validation of the RECONECT co-creation pathway will continue to be overseen by the project consortium who was dedicated to co-creation, social innovation and stakeholder's engagement in the process of NBS. The first lessons learned from co-creation in RECONNECT are provided separately [37].

**Table 10:** Validation of RECONECT's co-creation pathway/approach and the related tasks considered.

Task	How it is addressed by RECONECT co-creation pathway in an innovative way and validated by the RECONECT project partners
Create a common understanding of the co-creation concept and awareness of the importance of co-creation in NBS projects	RECONECT co-creation pathway provides the guiding principles of co-creation and explains how co-creation can help to promote social innovation and support the successful implementation of NBS. It addresses the connections between co-creation and social innovation specifically with regards to the co-assessment and planning, co-design, co-implementation, operation and maintenance, co-monitoring and evaluation of NBS.
Support a collaborative process with stakeholders in every phase / stage of an NBS project	RECONECT co-creation pathway presents a robust methodology for stakeholder engagement and participation in co-creation, which is introduced and mainstreamed throughout the NBS process and implemented across RECONECT's Collaborator and Demonstrator sites. The co-creation pathway consisting of seven steps to follow is accompanied by the description of every co-creation step. A **RECONECT multi-criteria decision-making matrix for co-creation** is introduced and its applicability is explained using several examples.
Lead to an appropriate selection of co-creation tools	A **RECONECT multi-criteria decision-making matrix for co-creation** supports the selection process of the most relevant tools according to the current needs, capacities, and goals of co-creation. The co-creation strategy is presented in a **seven steps co-creation framework** which navigates the practitioners during the co-creation process.
Provide toolboxes that are easy to apply thanks to practical instructions and background information	**Tollboxes for every stage of NBS development process** are presented, where goals, main steps/actions to take, stakeholders involved etc. are suggested along with the most appropriate tools to address the certain task and the main outcomes when applying the tools.
Be useful for training purposes concerning the co-creation of the NBS processes	An innovative **methodology to operationalize the co-creation of the NBS process** was tested at the RECONECT's Collaborator and Demonstrator sites. This entails designing a step-by-step co-creation pathway and indicators to assess the quality and progress throughout the process. It includes: - A seven steps co-creation pathway which navigates the practitioners during the whole co-creation process by explaining in detail how to start and what should be done at every step of co-creation. - Toolboxes for developing a co-creation at every stage of the NBS process (from co-assessment and planning to co-monitoring and evaluation) which provides a detailed set of steps for the strategic planning of a co-creation process. They are particularly useful for those who facilitate the co-creation strategy development process. - A RECONECT multi-criteria decision-making matrix for co-creation (in form of comparative chart) which helps to select the most relevant tool(s) according to the current needs, capacities and goals of co-creation. - Toolboxes for the co-creation of NBS at every stage of its process are useful for practitioners who would like to learn how to apply the tool in real-life settings by providing technical assistance as well as important notes that have emerged from previous experiences.

## Recommendations

Bearing in mind that the co-creation is an open process which runs throughout all NBS stages and according to the theory of change is subject to continuous transformation, we argue that co-creation process is not a constant process: it is instead a process that requires constant adaptation, changes and development of additional strategical elements. However, to set up such a process in practice, deciding which tools to use in order to engage stakeholders in a productive and just manner, is something that it is more difficult to acquire knowledge about. With this methodological paper, we aim to close this gap. The presented step-wise approach / pathway, based on the fundamental knowledge on co-creation along with the provided decision-making matrix of tools, enables the selection of appropriate co-creation tools according to the particular co-creation goal, resources and capacities, and explains in detail the practical realization process. It combines well-known business models with tools from design thinking that promote active participation by all relevant stakeholders. Some of the tools are common and actively used in the co-creation process of many NBS related projects. Other tools have not previously been applied to NBS; however, due to their high potential and expected value, they are also suggested for co-creation of NBS and have already been tested during RECONECT, receiving positive feedback and approval.

## Conclusions

The presented methodical approach explains how to apply the co-creation concept to designing and implementing NBS in rural and natural territories using the RECONECT seven-steps-pathway and provides a practical guide, including matrices of tools for co-creating NSB. By providing practical guidance on how to design, implement and facilitate the co-creation of NBS, it highlights in detail the importance of the co-creation approach for the whole NBS process and sets out specific activities (steps) for the implementation of NBS using a participatory approach which involves a diversity of stakeholders. It enables the raising of awareness around the NBS phenomenon and its co-benefits, enhancing knowledge co-production and learning across various social-ecological and cultural systems as well as influencing policy and governance in the field of NBS.

## Supplementary material *and/or* additional information

N/A.

## CRediT authorship contribution statement

**Dushkova Diana:** Conceptualization, Methodology, Data curation, Investigation, Resources, Validation, Writing – original draft, Writing – review & editing, Visualization, Supervision. **Kuhlicke Christian:** Conceptualization, Methodology, Supervision, Validation, Writing – review & editing, Project administration, Funding acquisition.

## Declaration of Competing Interest

The authors declare that they have no known competing financial interests or personal relationships that could have appeared to influence the work reported in this paper.

## Data Availability

No data was used for the research described in the article.
